# *Scutellariae Radix* and *Atractylodis Macrocephalae Rhizoma* pairs ameliorate preeclampsia via PI3K/AKT/eNOS pathway

**DOI:** 10.3389/fphar.2025.1614167

**Published:** 2025-07-11

**Authors:** Shujun Sun, Jie Li, Dingmei Qin, Xiaoxiao Yang, Huilin Zhang, Yue Yang, Mengmeng Li, Ruihua Jin, Jianye Dai, Yong Liu

**Affiliations:** ^1^ School of Biology and Food Engineering, Fuyang Normal University, Fuyang, China; ^2^ Anhui Rural Revitalization Collaborative Technology Service Center, Fuyang Normal University, Fuyang, China; ^3^ School of Pharmacy, Lanzhou University, Lanzhou, China; ^4^ Fuyang Women and Children's Hospital, Fuyang Normal University, Fuyang, China; ^5^ Anhui Province Key Laboratory of Embryo Development and Reproductive Regulation, Anhui Province Key Laboratory of Environmental Hormone and Reproduction, Fuyang Normal University, Fuyang, Anhui, China

**Keywords:** preeclampsia, scutellariae radix, atractylodis macrocephalae rhizoma, drug pairs, PI3K/Akt/eNOS pathway

## Abstract

**Introduction:**

Preeclampsia stands as a leading cause of maternal mortality. Scutellariae Radix and Atractylodis Macrocephalae Rhizoma (SA) is a commonly employed traditional Chinese medicine pair in the treatment of preeclampsia, yet the underlying mechanisms of their protective effects against preeclampsia remain elusive.

**Methods:**

In this study, the N- nitro-L-arginine methyl ester (L-NAME) was utilized to establish a preeclamptic pregnant mouse model, and the protective effects of SA were systematically evaluated. A comprehensive approach integrating network pharmacology analysis, quantitative proteomics, and in vitro experiments was adopted to probe into the relevant mechanisms.

**Results:**

It was demonstrated that SA could significantly ameliorate the systolic blood pressure, urinary protein levels, and pathological damage in the kidneys and placenta of mice. Moreover, in vitro experiments further validated the promoting effects of SA, baicalin, and atractylenolide I on the proliferation and migration of HTR-8/SVneo cells. Baicalin and atractylenolide I are the main active and quality control components of SA. Network pharmacology analysis, quantitative proteomics and molecular docking revealed that the PI3K/Akt pathway is a potential mechanism through which SA alleviates preeclampsia. Further investigations showed that SA could reverse L-NAME-induced inhibition of the PI3K/p-Akt/Akt signaling pathway, upregulating eNOS expression and ultimately alleviating vasoconstriction and other preeclampsia-related symptoms.

**Conclusion:**

SA has the capacity to improve preeclampsia-induced increases in blood pressure and urinary protein, holding promise as a novel strategy for the treatment of preeclampsia.

## 1 Introduction

Preeclampsia refers to the gestational disease with elevated blood pressure and proteinuria, accompanied by multiple symptoms after 20 weeks of gestation. If not treated in time, it may lead to increased seizures and mortality (Joshi and Joshi, 2017). At present, the global incidence is 1.5% and 16.7%, and results in 60,000 maternal deaths and >500,000 preterm birth worldwide each year. It was reported that preeclampsia was the second leading cause of maternal death (Chen et al., 2021; Brownfoot and Rolnik, 2024). Once preeclampsia or eclampsia is diagnosed, there is no completely curable drug at present. Clinically, pregnant with severe preeclampsia are usually recommended to terminate pregnancy. Therefore, it is highly urgent to develop therapeutics that directly target the disease (de Alwis et al., 2022).

Scutellariae Radix (SR) is the dry root of *Scutellaria baicalensis* Georgi. Atractylodis Macrocephalae Rhizoma (AM) is the dried rhizome of *Atractylodes macrocephala* Koidz. The compatibility of the two medicine is commonly used in clinical miscarriage prevention (Tian, 2006; Ming Dynasty Li Shizhen, 2011). Li (2021) reviewed the literature on the treatment of preeclampsia by traditional chinese medicine (TCM), and results showed that Scutellariae Radix and Atractylodis Macrocephalae Rhizoma (SA) were used more frequently in different TCM formulas. And in many prescriptions for the treatment of preeclampsia, SA exist in the form of drug pairs, such as Bazhen Yimu decoction (Ding and Shang, 2024). Li, (2019), used baicalin capsules combined with labetalol hydrochloride injection to treat severe preeclampsia, which has significant effect, can improve renal function and blood fluidity, and has high safety. Banxia Baizhu Tianma decoction has also been found to effectively improve the maternal and infant outcomes of severe preeclampsia in many clinical studies of traditional Chinese medicine. Liu et al. (2021), demonstrated that atractylenolide has anti-apoptotic and oxidative stress effects on htr-8/svneo in preeclampsia by activating MAPK/ERK signaling pathway. To date, no animal studies had been conducted to validate the efficacy of SA and its constituents for preeclampsia, and consequently, the underlying therapeutic mechanisms remain to be elucidated.

During normal pregnancy, increased activity of nitric oxide synthase (NOS) and production of nitric oxide (NO) contribute to vasodilation and reduce placental blood flow resistance (Mazloomi et al., 2021). Preeclampsia pregnant women have impaired NO bioavailability and signaling, decreased vasodilatory capacity, and increased placental vascular resistance, which in turn leads to hypertension. Endothelial nitric oxide synthase (eNOS) is activated by upstream p-Akt to release no with vasodilatory effect. Akt needs to be activated by phosphatidylinositol-3-kinase (PI3K) and its messenger molecules, so PI3k/Akt/eNOS pathway is a signal transduction pathway located in endothelial cells with important regulatory effects. Some researchers have also reported that the activation of PI3k/Akt/eNOS pathway is related to the proliferation and differentiation of human villous trophoblast cells involved in uterine spiral artery remodeling during pregnancy (Yang et al., 2013). In view of the wide application of SA on preeclampsia, it has high development value. The present study, for the first time, to observe the efficacy of SA on preeclampsia, and verify whether it plays a protective role through PI3K/Akt/eNOS pathway through *in vivo* and *in vitro* experiments.

## 2 Materials and methods

### 2.1 Materials and reagents

SR and AM were purchased from Bozhou Chinese Medicine Exchange in China, SR originates from Inner Mongolia (China) and AM originates from Jiangxi (China). N′-Nitro-L-arginine-methylesterhydrochloride (L-NAME) (51,298-62-5) was obtained from Yuanye Biotechnology (Shanghai, China). Human chorionic extrachorionic trophoblast cell (HTR-8/SVneo) (lh-h129) was purchased from BioHarbor Biotechnology Company (Shanghai, China). Antibodies against PI3K (YM3503), AKT (T0185), and P-AKT (YP0006) were purchased from ImmunoWay Biotechnology Company (Texas, United States). eNOS (AB300071) was obtained from Abcam (Cambridge, UK). C57BL/6J mice were purchased from Nanjing Jicui Pharmachem Laboratory Animal Technology (Nanjing, China). LY294002(HY-10108) was purchased from MedChemExpress (New Jersey, United States).

### 2.2 Preparation of SA extract

100 g SR and 150 g AM (traditional medicine ratio 2:3), add 8 times water after washing, decoct for 60 min, while constantly stirring, filtering, retain the decoction, repeat three times. The three decoctions were mixed, concentrated to the extract, and freeze-dried for 48 h to obtain the extract of SA for use.

This extract was preliminarily analyzed using the HPLC-Q Exactive-Orbitrap-MS (Thermo Fisher Scientific, Waltham, MA, United States). The chromatographic separation was performed using the Ultimate 3000 HPLC system (2.1 mm × 150 mm x 1.8 um) column (Welch). The mobile phase comprised (A) 0.1% methanol in pure waterand (B) methanol with gradient elution: 0–1 min, 98% (A); 1–5 min, 98%–80% (A); 5–10 min, 80%–50% (A); 10–15 min, 50%–20% (A); 15–20 min, 20%–5% (A); 20–27 min, 5% (A); 27–28 min, 5%–98% (A); 28–30 min, 98% (A). The flow rate was 0.3 mL/min and the column temperature was maintained at 40°C. The samples were maintained at room temperature and the injection volume was 5 μL. The Q Exactive mass spectrometer equipped with a heated electrospray ionization interface was operated under both electrospray ionization (ESI) negative and ESI positive modes. The instrument was calibrated with the calibration solutions provided by the manufacturer. Data were acquired using Compound Discoverer 3.3 software (Thermo Fisher Scientific, Waltham, MA, United States), and the preliminary sorting data were searched and compared in the database (mz Cloud). The other parameters were set as follows: The source parameters, spray voltage of 3.2 kV (+)/3.2 kV (−); capillary temperature, 300°C; auxiliary gas heater temperature, 350°C, sheath gas pressure, 40 arb; auxiliary gas pressure, 15 arb.

### 2.3 Animals experimental

L-NAME-induced hypertensive pregnant rats serve as a well-established animal model for preeclampsia. Pregnant mice were randomly divided into six groups (n = 6): control, model (L-NAME group), Aspirin (positive group, i. g, 15.2 mg/kg/d) + L-NAME, SA low-dose (i.g, 95 mg/kg/d) + L-NAME, SA middle-dose (i.g, 135 mg/kg/d) + L-NAME, SA high-dose (i.g, 175 mg/kg/d) + L-NAME. According to the Chinese Pharmacopoeia, the recommended daily dosages for adults are 3–10 g for Scutellaria baicalensis and 6–12 g for Atractylodes macrocephala (National Pharmacopoeia Commission, 2020). Based on the total amount of the compound formula and an extraction yield of 8.5%, the equivalent clinical extract dosage for adults was calculated to range from 95 to 270 mg/kg/day. Using the standard body surface area conversion factor of 9.01 to convert this dosage to mice, and considering toxicity data reported in the literature (Liu et al., 2014; Song et al., 2020; Yang et al., 2021), the low, medium, and high doses were finally set at 95, 135, and 175 mg/kg, respectively. Except for the control group, the other groups were given L-NAME (I.H, 50 mg/kg/d) subcutaneously daily from day 8 of pregnancy. The Aspirin and SA group were orally administered the respective compounds once daily, and the control and model groups received equal volume of saline. Each treatment was administered continuously on days 7 and 17 of pregnancy. Blood pressure was measured in each group on days 7 and 17 of pregnancy. Total protein was measured in urine on days 7 and 18 of pregnancy using the Total Urine Protein Test (UTP colorimetric method). Female rats were euthanized on day 18 of pregnancy, and placenta, fetus, and kidney tissue were collected. The study was approved by the Experimental Animal Center of Fuyang Normal University and conducted by the national and institutional guidelines regarding animal experiments. The Institutional Animal Care Committee of the university reviewed and approved the study protocol (Approval Number, K-2022-0529-1).

### 2.4 Network pharmacology analysis

The screening of active compounds and identification of targets involved utilizing the Traditional Chinese Medicine Systems Pharmacology Database (TCMSP, http://lsp.nwu.edu.cn/) and the Chinese Academy of Sciences chemistry database (CASC, http://www.organchem.csdb.cn/scdb/main/) to identify active compounds from SR and AM, focusing on those with oral bioavailability (OB) ≥ 20% and drug-likeness (DL) ≥ 0.1, along with quality control compounds. Subsequently, details on human genes associated with preeclampsia were gathered from three databases: the Comparative Toxicogenomics Database (CTD, https://www.pharmgkb.org/), OMIM (https://www.omim.org/), and GeneCards (https://www.genecards.org/), with gene identifiers converted into official gene symbols using an R-script linked to PubMed (https://www.ncbi.nlm.nih.gov/gene/), ensuring only human genes were included for further analysis. Common target genes for the drugs and diseases were then obtained, leading to the construction of a network of compound-common targets, while protein-protein interaction (PPI) networks for the compound targets and preeclampsia targets were visualized using Cytoscape 3.7.1 software. Finally, bioinformatic analysis was performed through KEGG enrichment analysis using the Database for Annotation, Visualization, and Integrated Discovery (DAVID, https://david.ncifcrf.gov, v6.8), identifying pathways with significant changes (P < 0.05) for further analysis, selecting genes that significantly regulated these pathways for gene-pathway network analysis, and identifying key target genes from the resulting network.

### 2.5 Molecular docking

The PDB file of PI3K was download from database (https://bitterdb.agri.huji.ac.il/dbbitter.php), Atractylenolide I and Baicalin mol2 files downloaded in PubChem database (https://pubchem.ncbi.nlm.nih.gov/), analysis software for AutoDockTools-1.5.7, visualization tools for PyMOL-2.6.0.

### 2.6 Hematoxylin and eosin (HE) staining

To assess histopathological changes, placenta and kidney tissue were fixed in 4% formaldehyde and embedded in paraffin. Sequential serial sections were deparaffinized and stained with hematoxylin and eosin (HE) for histological examination.

### 2.7 Western blotting analysis

The samples were lysed in a RIPA buffer solution involving 1% protease inhibitor. After centrifugating, supernates were gathered. Determination of protein level was conducted via the BCA protein assay kit. The denatured protein was isolated via 10% SDS-PAGE and shifted to PVDF membrane through electrophoresis. Membranes were blocked with 5% skim milk and then co-incubated overnight under 4°C with primary antibodies (including PI3K, AKT, P-AKT and eNOS). After washing, membranes were incubated with secondary antibodies for 2 hours at ambient temperature. Quantification of the protein bands was performed via ImageJ.

### 2.8 Cell proliferation

HTR–8/SVneo cells were cultured in RPMI–1640 with 10% FBS, at 37°C with 5% CO_2_. Cells were plated at 5 × 10^4^ cells/well in 96-well plates for 24 h and then treated with L-NAME for 2 h. Each well was added SR and atractylodis extract, baicalin, atractylenolide I, and then added MTT after 12 h and incubated at 37°C for another 5 h. The formazan crystals were dissolved in DMSO. The absorbance was measured at 490 nm.

### 2.9 LDH secretion of cell

Cells were plated at 5 × 10^4^ cells/well in 96-well plates for 24 h and then treated with L-NAME for 2 h. Each well was added SA, baicalin, atractylenolide I. The cell supernatant was aspirated and centrifuged to remove solid impurities from the supernatant. The absorbance of NADH was measured by spectrophotometer at 340 nm at room temperature.

### 2.10 Cell migration

Cells were plated at 5 × 10^4^ cells/well in 6-well plates for 48 h and then scratches were made on the bottom of the dishes with a lance tip. The well plates were divided into six groups, excluding the control group, the other five groups were added with the same concentration of L-NAME in each well, incubated in the incubator for 2 h. Each well was added SA extracts, baicalin, atractylenolide I. and compared of scratches photographed under microscope at 0 h, 12 h, and 24 h.

### 2.11 Immunofluorescence

Cells were plated at 5 × 10^4^ cells/well in 6-well plates with spread for 48 h. Cells were fixed by soaking in ethanol and permeabilized using Triton X-100. The cells were restored to room temperature and incubated with appropriate fluorescence-conjugated secondary antibodies and DAPI. The images were captured with a Nikon confocal microscope and calculated with NIH ImageJ software.

### 2.12 Quantitative proteomic spectrometry detection

The method of stable isotope dimethyl labeling was used to study the quantitative proteomic analysis of placenta. Analysis was performed based on published technical protocols using a Q-Exactive Orbitrap mass spectrometer coupled to an Ultimate 3000 LC system (Zhu et al., 2024). Proteins with an average ratio ≥1.5 or ≤0.67 in all samples were selected for KEGG pathway analysis and GO analysis by DAVID (https://david.ncifcrf.gov/). The selected proteins were imported into the STRING (http://stringdb.org/) database to construct a protein-protein interaction (PPI) network.

### 2.13 Statistical analysis

SPSS 22.0 (IBM Corp., Armonk, NY, United States) and GraphPad Prism 8.2.0 (GraphPad Software Corp., San Diego, California, United States) were used for statistical analysis. Statistical analysis was performed with one-way ANOVA with Dunn’s test for multiple comparisons, while unpaired Student’s t-test for two individual comparisons. p < 0.05 is considered significant. Mean ± standard deviation (SD) is the expression of the data. The proteomic data were deposited to the ProteomeXchange consortium (https://proteomecentral.proteomexchange.org) via the iProX partner repository (Ma et al., 2019; Chen et al., 2022) with the dataset identifier PXD038535.

## 3 Results

### 3.1 Analysis of scutellariae radix and atractylodis macrocephalae rhizoma

**FIGURE 1:**
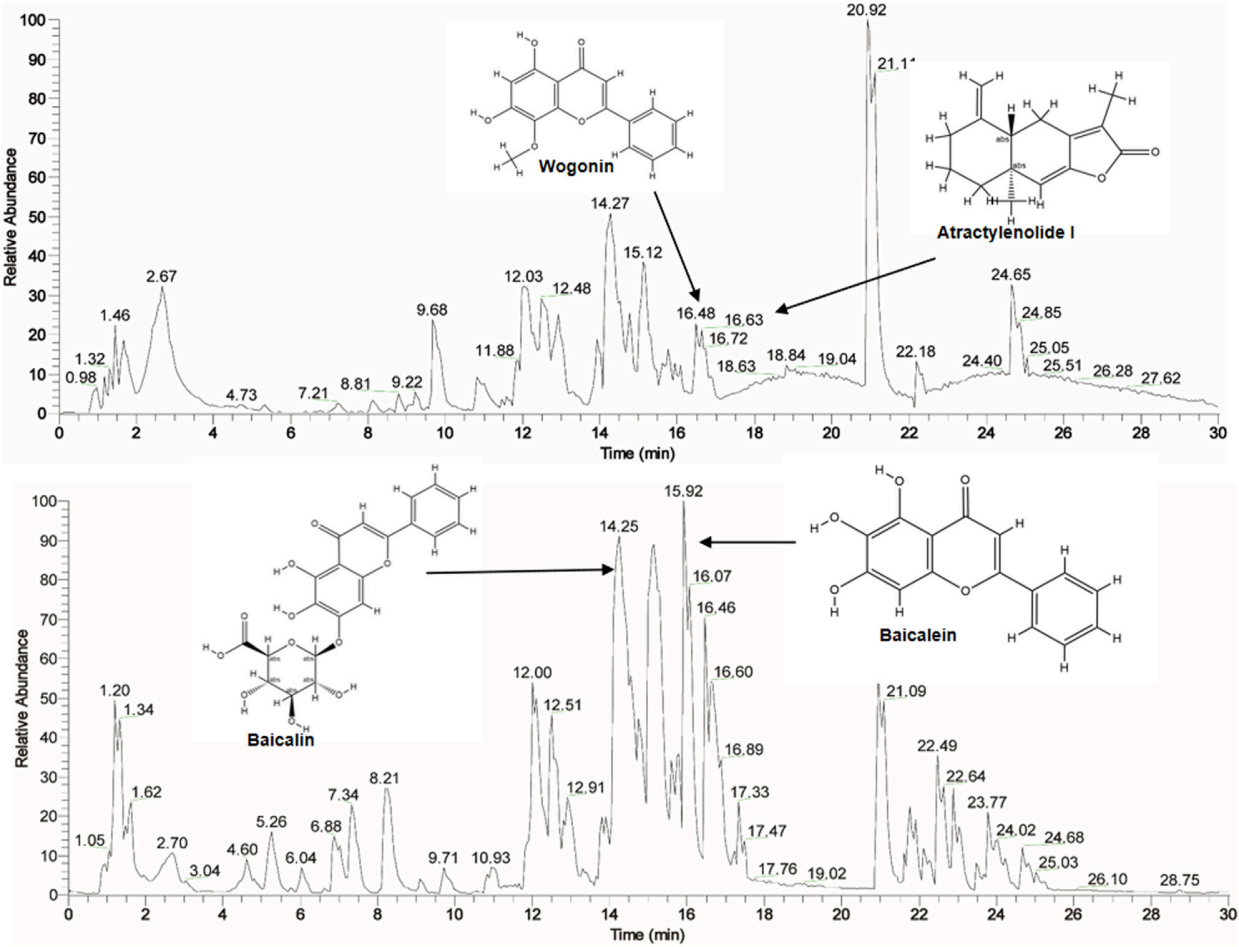
The fingerprint of SA by HPLC-Q Exactive-Orbitrap-MS instrument.

This extract was preliminarily analyzed using the HPLC-Q Exactive-Orbitrap-MS. The data acquired from HPLC-Q Exactive-Orbitrap-MS instrument were initially processed using Compound Discoverer 3.3 (CD 3.3, Thermo Fisher) and subsequently compared against the mzCloud database. In total, 636 compounds were identified in mz Cloud, with 132 compounds achieving a comprehensive score of mz Cloud best match exceeding 90 (Supplementary Table S1). The fingerprint of SA is attached in Figure 1. The major bioactive components commonly identified in *S. baicalensis* and *Atractylodis Macrocephalae Rhizoma*, such as Wogonin, Atractylenolide I, Baicalin, and Baicalein, were clearly annotated in the fingerprint chromatogram (Figure 1).

### 3.2 SA ameliorate preeclampsia

**FIGURE 2:**
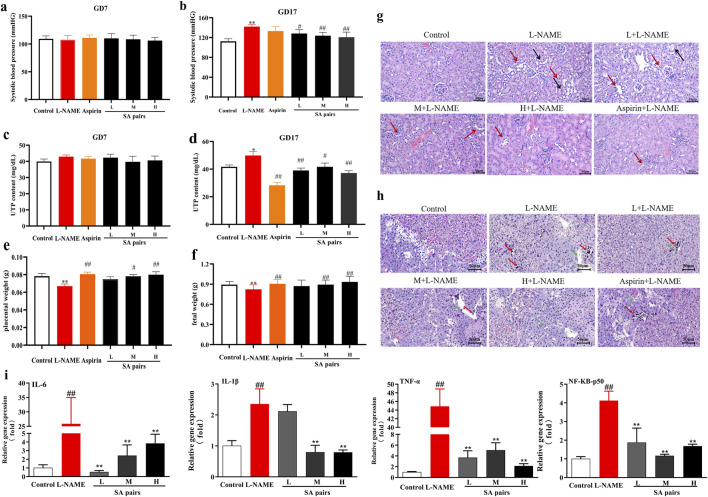
Protective effects of SA preeclampsia induced by L-NAME (n = 6). **(a,b)**, blood pressure. **(c,d)**, urine protein (UTP). **(e,f)**, placenta (or fetal) weight. **(g)**, HE staining of kidney (Black arrow:the renal tubules were severely vacuolated, red arrow: a large number of nuclei were exposed and shed). **(h)** placenta tissue (Red arrows:necrotic foci, green arrows:calcified foci. The magnification of the HE pathological staining images is set at 20 times). **(i)** mRNA level of inflammatory factors in placenta. ^**^p < 0.01, Compared with Control group, ^#^p < 0.05, ^##^p < 0.01, Compared with L-NAME group.

This part first evaluated the effects of SA on preeclampsia. Results showed that the systolic blood pressure and urinary protein levels of mice in model group were significantly higher than those in control group after 10 days of L-NAME administration (i.e., the 17th day of pregnancy (gd17)). However, SA treatment could decrease the systolic blood pressure and urinary protein of mice (Figures 2a–d). The organ index test showed that the weight of placenta and fetal rats in the SA treatment group was significantly higher than that in the model group (Figures 2e,f), suggesting that they can improve the growth of placenta and fetus. Further HE staining showed that the renal tubular epithelial cells in the control group were round and plump, arranged regularly, and the renal tubular cells were coated with nuclei. In the model group, the renal tubules were severely vacuolated (black arrow), a large number of nuclei were exposed and shed (red arrow), and some glomeruli were swollen. In the SA treatment group, vacuolization of renal tubules was reduced, and only a few nuclei were exposed and shed (red arrow) (Figure 2g). HE staining of placental tissue showed that in the control group, the boundaries of each layer were clear, the trophoblast cells were arranged regularly, the morphology was normal, and a large number of red blood cells could be seen. In the model group, the boundary between placental trophoblast layer and labyrinth layer was unclear, necrotic foci (red arrows) were seen in many places, trophoblast nuclei were pyknotic, deeply stained and fragmented, calcified foci (green arrows) were seen in necrotic foci, and the surrounding trophoblasts were arranged irregularly; Red blood cell content decreased. After treatment with SA, the above injury can be significantly improved (Figure 2h). As inflammation is a critical pathogenic mechanism and physiological manifestation of preeclampsia, we assessed the activation of the NF-κB pathway by analyzing the mRNA levels of key inflammatory factors, including IL-6, IL-1β, TNF-α, NF-κB-p50, and NF-κB-Rel A. The results demonstrate that SA can significantly modulate the expression levels of these inflammatory factors in the placental tissues of preeclampsia mice (Figure 2i).

### 3.3 SA, baicalin and atractylenolide I promote the proliferation and migration of HTR-8/SVneo cell

**FIGURE 3:**
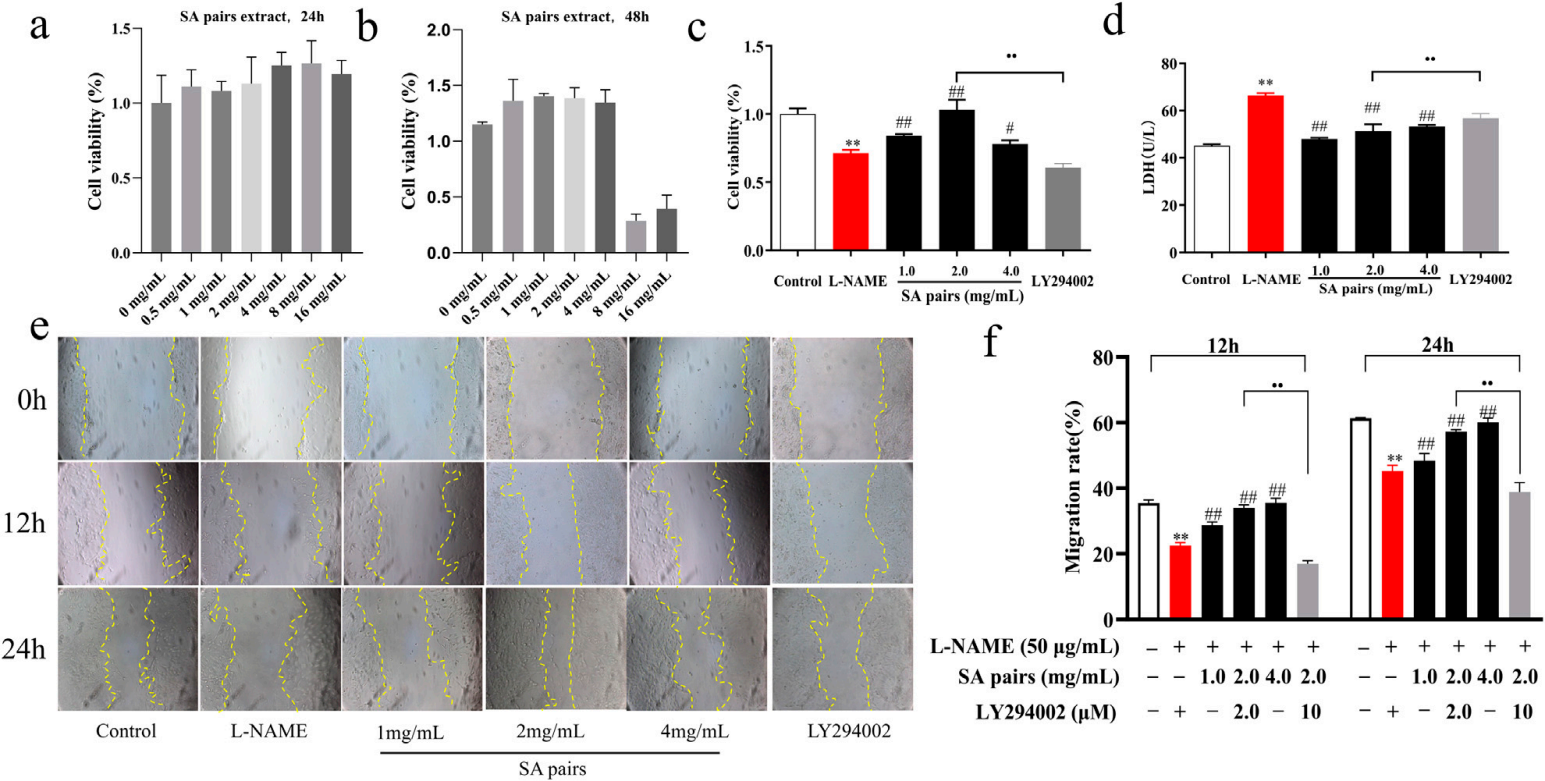
Effects of SA on proliferation and migration of HTR-8/SVneo cell (n = 3). **(a)** and **(b)** Dose-response cell vability experiments, **(c)** Cell proliferation rate, **(d)** Secretion rate of LDH, **(e)** and **(f)** Cell migration (The magnification is set to 10x). **p < 0.01, Compared with Control group, ^##^ *p* < 0.01, Compared with the L-NAME group, ••*p* < 0.01, Ly294002 group compared with the SA Middle dose group (2 mg/mL), 12h, The migration rate at 12 hours of drug treatment, 24h, The migration rate at 24 hours of drug treatment.

After clarifying the protective effect of SA on preeclampsia, this part continued to observe its protective effect using *HTR-8/SVneo* cells. To demonstrate the pharmacological rationale for selecting specific concentrations, dose-response cell vability experiments firstly were conducted. SA pairs is safe for HTR-8/SVneo cells within the dose range of 0–4 mg/mL, as shown in Figure 3a. Such three doses (1,2,4 mg/mL)were selected for experiments of cell proliferation and migration. The results showed that compared with the control group, L-NAME treatment could lead to decreased cell proliferation, increased LDH secretion, and slowed wound healing. When treated with SA, compared with the L-NAME group, different doses of SA can improve the cell proliferation rate, reduce LDH secretion, and promote the migration level. After adding the PI3K inhibitor LY294002 to the HTR-8/SVneo cells, it was found that the promoting proliferation effect of SA on L-NAME-stimulated HTR-8/SVneo cells was significantly weakened (Figures 3c–f).

**FIGURE 4:**
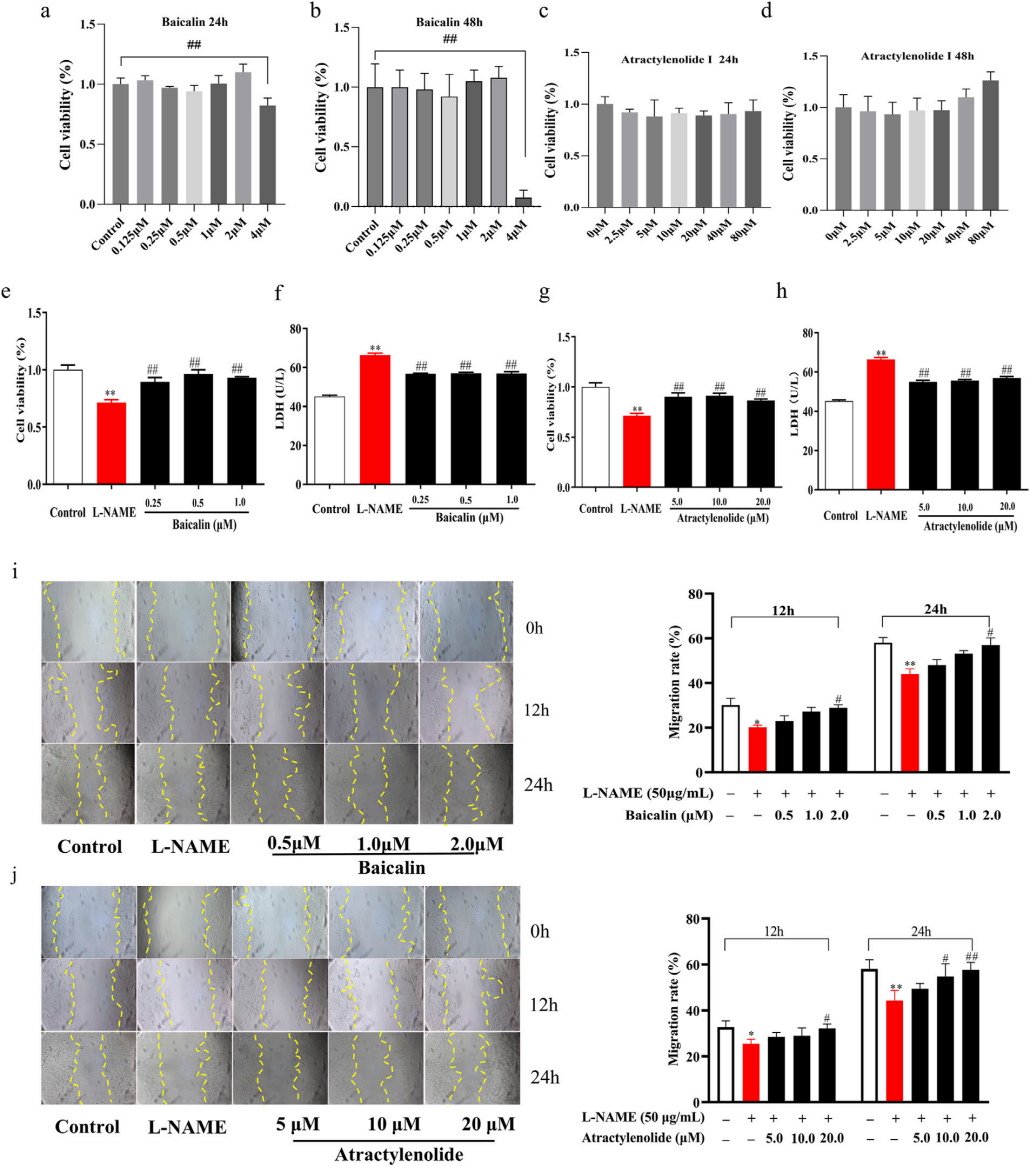
Effects of baicalin and atractylenolide I on proliferation and migration of HTR-8/SVneo cell (n = 3). **(a–d)**, Dose-response cell vability experiments for baicalin and atractylenolide I, **(e)** and **(g)** Cell proliferation rate, **(f)** and **(h)** Secretion rate of LDH supernatant, **(i)** Cell migration under the treatment of baicalin (The magnification is set to 10x), **(j)** Cell migration under the treatment of atractylenolide I. **p < 0.01, Compared with Control group, ^##^ *p* < 0.01, Compared with the L-NAME group, 12h, The migration rate at 12 hours of drug treatment, 24h, The migration rate at 24 hours of drug treatment.

In order to further verify the effect of SA on the proliferation and migration of HTR-8/SVneo cell, we selected baicalin and atractylenolide, the main active and quality control components of SA, respectively. Results showed that its effect on the proliferation, LDH secretion and migration of HTR-8/SVneo cells was the same as that of SA (Figure 4).

### 3.4 PI3K/AKT is potential pathway for SA

**FIGURE 5:**
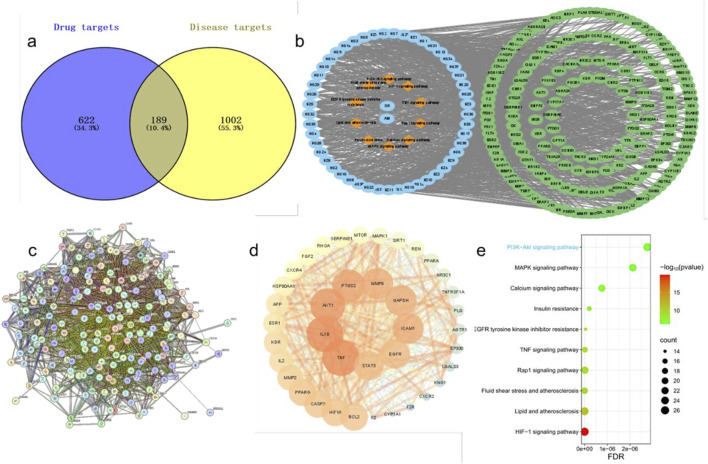
Potential target prediction of SA in preeclampsia treatment and construction of PPI and drug-disease network. **(a)**, Venn diagram of active ingredient targets and disease targets. **(b)**, Drug-active ingredient-target-pathway network diagram. **(c)**, PPI protein interaction network diagram. **(d)**, The visualized PPI network of the intersecting targets. **(e)**, The bubble diagram of top 10 KEGG pathway.

Through the Traditional Chinese Medicine Systems Pharmacology Database and Analysis Platform (TCMSP), we identified 143 chemical constituents in SR and 55 in AM. By applying the selection criteria of oral bioavailability (OB) ≥ 20% and drug-likeness (DL) ≥ 0.1, along with the inclusion of pharmacologically active components commonly recognized in SR and AM, such as baicalin, baicalein, atractylenolide I, atractylodin, and atractylolide III, we ultimately obtained 38 active constituents from SR and 11 from AM. The effective components’ targets were mapped against disease targets using Venny 2.1.0, yielding 189 common genes (Figures 5a,b). A protein-protein interaction (PPI) network diagram was constructed for these 189 effective targets. Using Cytoscape software, 39 core targets were identified, with key target genes including TNF, IL1B, AKT1, and PTGS2, among others (Figures 5c,d). The aforementioned 189 targets were imported into the DAVID database for KEGG analysis. Given that this study primarily focuses on preeclampsia, signal pathways unrelated to the pathogenesis of this disease, such as those associated with cancer, were excluded. The top 10 signal pathways were visually processed, which mainly included the TNF signaling pathway, the PI3K/AKT signaling pathway, and the Relaxin signaling pathway (Figure 5e). Considering the significant role of the PI3K/AKT signaling pathway in the development of vascular diseases, further validation of this pathway will be pursued in subsequent studies.

### 3.5 Quantitative proteomics verified that PI3K/AKT/eNOS pathway is closely related to SA ameliorate preeclampsia

To comprehensively elucidate the biological reaction mechanisms, including pathogenesis and pharmacodynamic effects. Proteomes extracted from placental tissues of pregnant rats in control, model, and medium-dose SA treatment groups were subjected to stable isotope dimethyl labeling followed by HPLC-MS/MS analysis.

**FIGURE 6:**
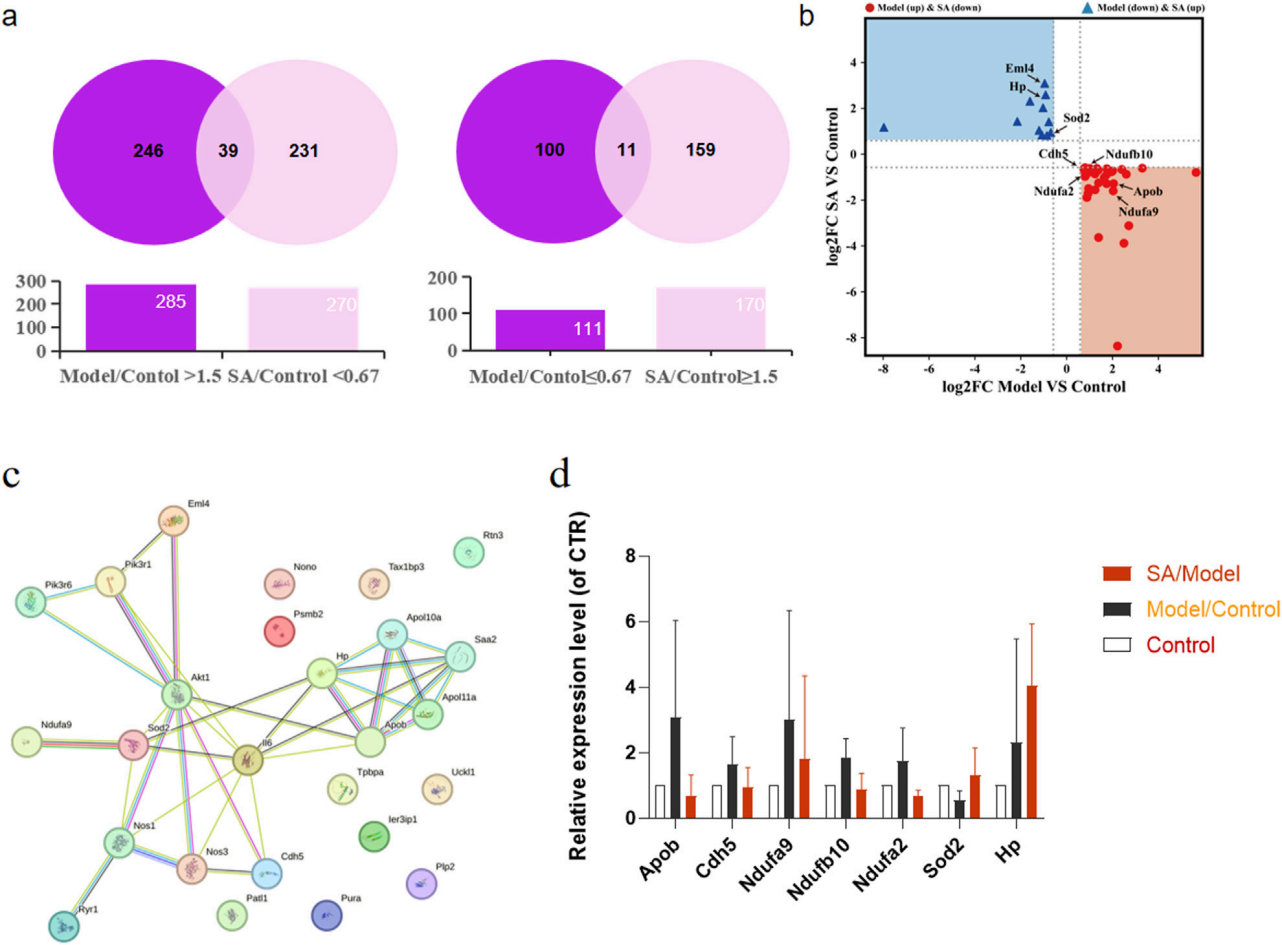
Quantitative proteomics experimental. Pregnant rat placenta of control group, model group and SA-pairs medium group (n = 3) were detected by HPLC-HRMS analysis. **(a)**: Venn diagrams illustrating proteins reversed by SA treatment included: (left) 39 targets suppressed after being pathologically overexpressed, and (right) 11 targets restored after pathological suppression. **(b)**: A four-quadrant plot analyzing potential drug target proteins identified in venn diagrams. **(c)**: Protein-protein interaction (PPI) network illustrating the connections between the 50 candidate proteins and the PI3K signaling pathway. **(d)**: Bar charts prominently highlighting the expression levels of eight key PI3K pathway-related proteins.

Protein screening criteria were established as follows: (1) proteins with ≤0.67-fold change in normal vs. model group and ≥1.67-fold change in SA-treated vs. model group; (2) proteins with ≥1.67-fold change in normal vs. model group and ≤0.67-fold change in SA-treated vs. model group. The analysis revealed 285 significantly upregulated and 111 downregulated proteins in the model group. SA treatment resulted in the regulation of 270 downregulated and 170 upregulated proteins, which was illustrated by venn diagrams in Figure 6a (The tables of the top 20 differentially expressed proteins, including their fold changes and adjusted P-values, were listed in Supplementary Table S2, S3), with 39 targets suppressed after being pathologically overexpressed, and (right) 11 targets restored after pathological suppression. A four-quadrant plot was used to analyze the potential drug target proteins identified in venn diagrams (Figure 6b).

Furthermore, we conducted protein-protein interaction analysis using the STRING database for the 50 candidate proteins along with key pathway components PI3K, Akt, and eNOS. The network analysis identified eight proteins interacting with the PI3K/Akt/eNOS pathway, among which superoxide dismutase-2 (SOD2), apolipoprotein B (ApoB), cadherin-5 (CDH5), and echinoderm microtubule-associated protein-like 4 (EML4) demonstrated direct interactions (Figure 6c). The raw data of the eight key proteins identified in the PPI network were organized into bar charts to visually demonstrate the reversing effect of SA on their expression levels (Figure 6d). These findings suggest that SA pairs may exert therapeutic effects on preeclampsia through the PI3K/Akt/eNOS signaling pathway.

### 3.6 SA, baicalin, and atractylenolide I activating PI3K/AKT/eNOS pathway

**FIGURE 7:**
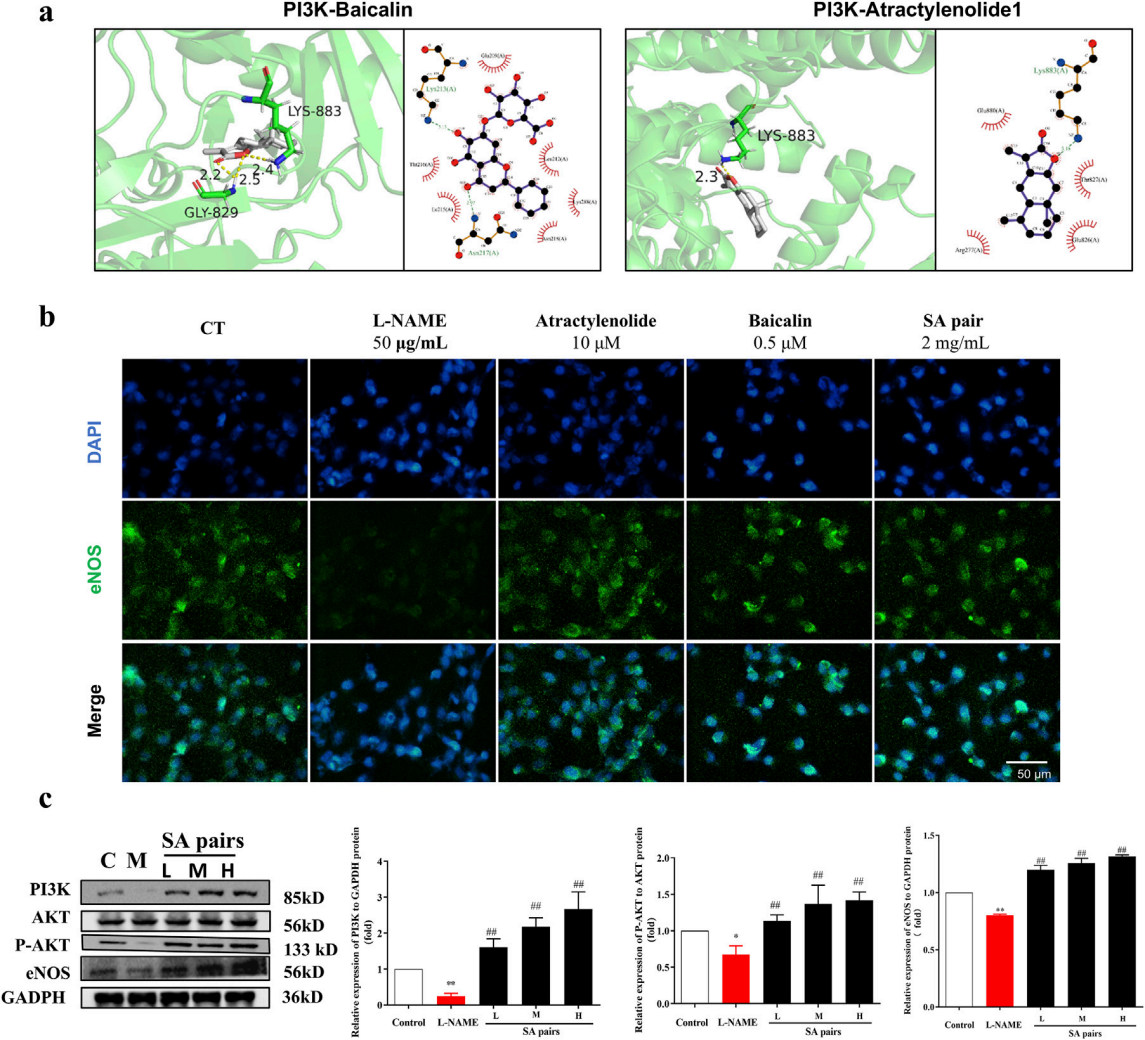
Influence of SA treatment on the expression of PI3K/AKT/Enos *in vivo*. **(a)** Molecular docking technology suggests that both baicalin and atractylenolide I can interact with PI3K. **(b)** HTR-8/SVneo cell was stained with antibodies against eNOS (green, n = 3). **(c)** Western blot bands and histograms for PI3K, P-AKT and eNOS in the *in invo* experiment (n = 3). *p < 0.05, **p < 0.01, Compared with Control group, ^##^p < 0.01, Compared with the L-NAME group.

**FIGURE 8:**
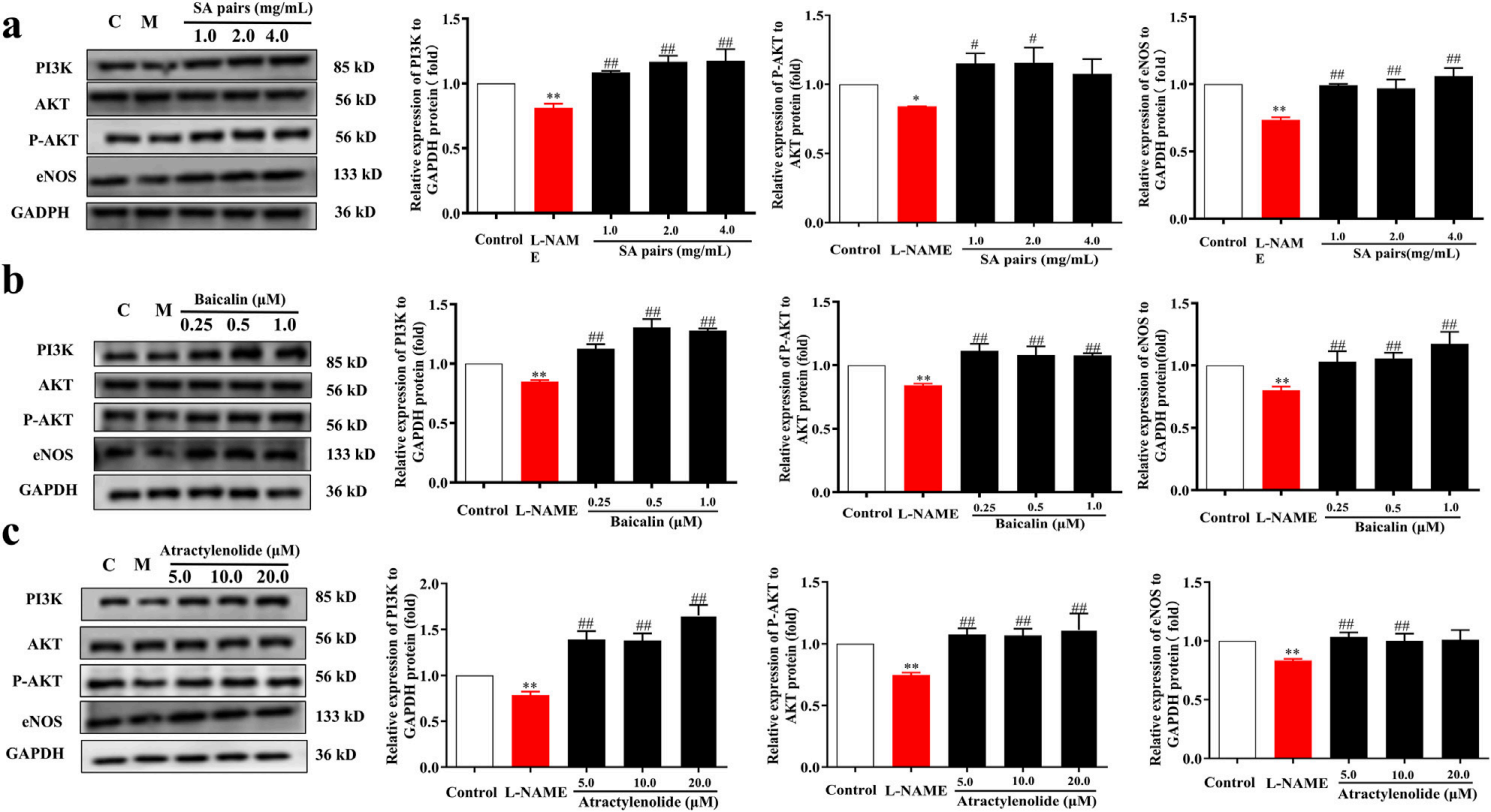
Influence of SA, baicalin and atractylenolide I treatment on the expression of PI3K/AKT/eNOS in vitro (n = 3). Western blot bands for PI3K, P-AKT and eNOS. **(a)** SA, **(b)** baicalin, **(c)** atractylenolide I. ^*^p<0.05, ^**^p<0.01, Compared with Control group, ^##^p<0.01, Compared with the L-NAME group.

Molecular docking results further showed that atractylenolide I and baicalin had a good binding activity with PI3K, with the binding energy were −5.72 and −3.6 kcal·mol^-1^, respectively ((Figure 7a). atractylenolide I and baicalin can form hydrogen bonds with Thr-827, Glu-800, Glu-209, Lys-213, Lys-883, etc. amino acid residues (Figure 7a). Besides, the expression levels of PI3K, p-Akt/Akt and eNOS in the model group were significantly downregulated, while the expression levels of them were significantly upregulated after treatment with SA in the *in vivo* experiment (Figure 7c). In the *in vitro* experiment, immunofluorescence staining results showed that the fluorescence intensity of eNOS in model group decreased, while the fluorescence intensity of baicalin, atractylenolide I, and SA treatment groups increased, suggesting that the SA treatment can reverse the reduction of eNOS expression caused by L-NAME (Figure 7b). Western Blot analysis showed that baicalin, atractylenolide I, and SA could reverse the decreased expression of PI3K, p-Akt/Akt and eNOS caused by L-NAME (Figures 8a–c). These results also showed that SA could upregulate PI3K, p-Akt/Akt pathway, increase eNOS expression, ultimately alleviate hypertension in the PE model.

## 4 Discussion

**FIGURE 9:**
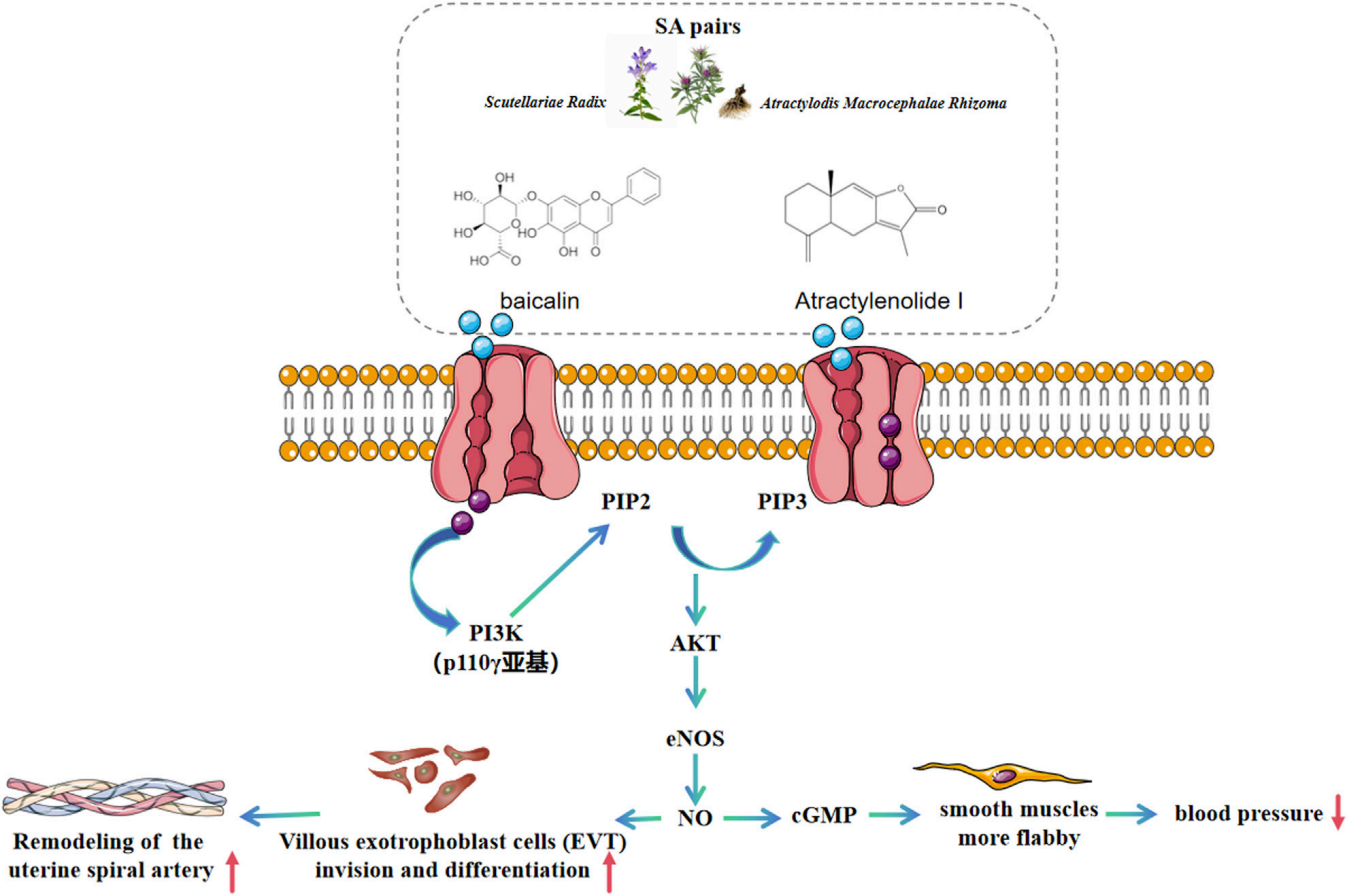
Therapeutic mechanism schematic of SA on preeclampsia based on PI3K/AKT/eNOS pathway.

In this study, we demonstrated that SA could effectively improve preeclampsia in mice. It significantly reduced the systolic blood pressure and urinary protein levels in preeclamptic pregnant mice. Moreover, SA ameliorated the pathological conditions of the placental tissue. Additionally, it also capable of facilitating the proliferation and migration of HTR-8/SVneo stimulated by L-NAME. Our findings revealed that the underlying mechanism is associated with the activation of the PI3K/Akt/eNOS pathway (Figure 9). These results provide a solid foundation for exploring SA as a potential therapeutic strategy for preeclampsia.

In prior studies, researchers have investigated the roles of baicalin and atractylenolide in the treatment of preeclampsia (PE). Paudel et al. demonstrated that baicalin reduces the migration of vascular smooth muscle cells (VSMCs) by inhibiting the expression of adhesion molecules and mitigates VSMC apoptosis through the modulation of reactive oxygen species (ROS) activity. Furthermore, studies have shown that baicalin exhibits substantial antihypertensive effects in PE rat models and enhances liver and kidney function (Paudel and Kim, 2020). Liu et al. additionally reported that atractylenolide provides protective effects in PE by activating the MAPK/ERK signaling pathway, which suppresses apoptosis and oxidative stress in HTR-8/SVneo cells (Liu et al., 2021). Based on these findings, our study extends this understanding by confirming the therapeutic effects of SA on PE via the PI3K/AKT/eNOS signaling pathway. In comparison to previous research, our study not only verifies the overall efficacy of these herbal components but also elucidates their molecular mechanisms, particularly the activation of the PI3K/AKT/eNOS pathway in alleviating the pathological conditions associated with PE, thereby offering novel theoretical insights for targeted PE therapy.

PI3K/Akt/eNOS pathway is a signal transduction pathway that plays an important regulatory role in endothelial cells (Ozmen et al., 2022). PI3K inhibitors have been extensively utilized as therapeutic strategies for various diseases, including cancer and fibrosis, as highlighted in numerous research reports. According to the review by Wang et al. (Wang et al., 2022), compounds such as astragaloside IV, hypericin, ligustrazine, and quercetin exert their effects through direct binding to PI3K and inhibiting its activity—a well-established mechanism that aligns with most pharmacological strategies targeting this pathway. Notably, this study revealed that SA pair can treat preeclampsia by activating the PI3K pathway and may directly interact with the PI3K protein. Although this conclusion deviates from traditional understanding, Knight et al. (Knight et al., 2006) unexpectedly discovered during inhibitor screening that PI-103 acts as a potent activator of the p110γ/p84 complex at specific concentrations. This finding provides robust support for the conclusion proposed in this article: that SA pair and its monomer compounds alleviate preeclampsia by activating the PI3K pathway.

The quantitative proteomic analysis of fetal placenta samples in this study revealed aberrant expression of proteins associated with the PI3K/Akt/eNOS pathway, including Superoxide dismutase-2 (SOD2), Haptoglobin (HP), apolipoprotein B (ApoB), Cadherin-5 (CDH5), and Echinoderm Microtubule-Associated Protein-Like 4 (Eml4). It is noteworthy that the changing trends of these proteins observed in preeclampsia mice align closely with those documented in previous studies. For example, SOD2 functions as an antioxidant enzyme in the mitochondrial matrix, protecting cells from oxidative damage by scavenging superoxide radicals (Fridovich I., 1995; Miao and St Clair, 2009). Roland et al. demonstrated that, in non-labor (cesarean section) conditions, the expression levels of SOD1 and SOD2 in the placental villi of preeclamptic mice were significantly reduced compared to the control group (p < 0.049) (Roland et al., 2010). ApoB serves as the core structural protein of lipoproteins, including very low-density lipoprotein (VLDL) and low-density lipoprotein (LDL), mediating cholesterol transport and cellular uptake (Clarke et al., 2023). Numerous studies have shown that ApoB levels were significantly increased in the blood of patients with preeclampsia (P < 0.05) (Dong et al., 2021; Khaire et al., 2021; Catarino et al., 2008). In this study, we observed that ApoB expression was downregulated in the placentas of preeclampsia mice but was upregulated following treatment with SA pairs. These findings suggest that SA pairs may regulate cholesterol metabolism in preeclampsia mice by modulating ApoB expression. CDH5 (VE-cadherin) is an endothelial-specific cell adhesion molecule that plays a critical role in regulating vascular barrier integrity and permeability (Dejana and Vestweber, 2013). Zhou et al. discovered that antibodies targeting VE-cadherin (Also known as CDH5 (Sauteur et al., 2014)) reduced the invasiveness of CTBs, suggesting a positive correlation between CDH5 and CTB cell invasion. However, the expression pattern of CDH5 in different types of cells within placental tissue is inconsistent. For instance, endothelial cells exhibit higher CDH5 content compared to human chorionic trophoblast cells (Varshavsky et al., 2020). Therefore, these findings are not entirely consistent with the present study, which revealed upregulation of CDH5 in the placentas of hypertensive pregnant rats. Further investigation is warranted to determine whether there exists a negative feedback mechanism leading to CDH5 upregulation in L-NAME-stimulated human chorionic trophoblast cell proliferation and migration. MAP4 functions as a microtubule-stabilizing protein, modulating cell division and migration by maintaining cytoskeletal dynamics (Fang et al., 2011). Thapa et al. found that MAP4 (alternate name EML4) controls the interaction of PI3Kα with activated receptors at endosomal compartments along microtubules. Loss of MAP4 results in the loss of PI3Kα targeting and loss of PI3K-Akt signaling downstream of multiple agonists (Thapa et al., 2020), which was consistent with the result of this article that the EML4 was downregulated and PI3K/Akt/eNOS pathway was inhibited in the preeclampsia model group. The literature supports consistent changes in ApoB, Sod2, and EML4 trends with the results of this study, confirming a potential relationship between the therapeutic effects of SA on preeclampsia and the PI3K/Akt/eNOS pathway. Therapeutic mechanism schematic of SA on preeclampsia based on PI3K/AKT/eNOS pathway is illustrated in Figure 9.

Although this study demonstrates promising results regarding the Scutellariae Radix-Atractylodis Macrocephalae Rhizoma pair’s therapeutic potential for preeclampsia through the PI3K/AKT/eNOS pathway, several limitations must be acknowledged. The findings are currently restricted to animal models and *in vitro* studies, lacking clinical validation of safety and efficacy in humans. Moreover, PE’s complex pathogenesis likely involves additional signaling pathways beyond PI3K/AKT/eNOS, and the potential synergistic effects of other active components in the herb pair remain unexplored. Future research should prioritize clinical trials while expanding investigations to uncover other relevant molecular mechanisms. Comprehensive phytochemical analysis of the herb pair’s bioactive components, along with optimization of dosage regimens, will be essential for translating these findings into effective clinical applications with minimized adverse effects.

## 5 Conclusion

SA could ameliorate L-NAME induced preeclampsia, which also improved the proliferation and migration rate of L-NAME-stimulated HTR-8/SVneo, and we demonstrated that SA can ameliorate preeclampsia by activating the PI3K/AKT/eNOS pathway, as confirmed through quantitative proteomics and molecular biology techniques. SA is expected to provide a new strategy for the treatment of preeclampsia.

## Data Availability

The original data presented in the study are included in the article/Supplementary Material, further inquiries can be directed to the corresponding authors.
