# Supplementary material for: Scutellariae Radix and Atractylodis Macrocephalae Rhizoma pairs ameliorate preeclampsia via PI3K/AKT/eNOS pathway
Source: Front Pharmacol. 2025 Jul 11;16:1614167. doi: 10.3389/fphar.2025.1614167 (PMC12290292; doi:10.3389/fphar.2025.1614167)
Supplement: Supplementary file 1 [file Table1.pdf]

Supplementary materials

Table S1 Structural characterization of the identified compounds

NO	Name	Formula	m/z	RT [min]	mzCloud Best Match	Area
1	Ornithine	C5 H12 N2 O2	131.08127	1.185	99.3	1046633396
2	L-Histidine	C6 H9 N3 O2	154.06102	1.204	97.7	269981977.5
3	DL-Arginine	C6 H14 N4 O2	175.11882	1.215	94.6	49202148198
4	Choline	C5 H13 N O	104.10737	1.349	97.5	4408868377
5	D-(+)-Maltose	C12 H22 O11	365.10498	1.446	95.8	700237824.3
6	Maltotetraose	C24 H42 O21	689.21021	1.451	94.8	132919009.4
7	Trigonelline	C7 H7 N O2	138.0549	1.472	99.1	630945773.6
8	4-Guanidinobutyric acid	C5 H11 N3 O2	146.09232	1.479	93.5	1151162091
9	Acetylarginine	C8 H16 N4 O3	217.12949	1.513	99	227702464.5
10	D-(-)-Quinic acid	C7 H12 O6	191.05505	1.519	97.2	2778856282
11	4-ethyl-5-(10H-phenothiazin-10-ylmethyl)-2,4-dihydro-3H-1,2,4-triazole-3-thione	C17 H16 N4 S2	363.06842	1.525	94	486283519.5
12	Adenine	C5 H5 N5	136.06181	1.577	99.6	1169685015
13	Asparagine	C4 H8 N2 O3	133.06084	1.583	95.4	1865622415
14	Glucose 1-phosphate	C6 H13 O9 P	261.03687	1.765	99.3	856064699.7
15	DL-Stachydrine	C7 H13 N O2	144.10199	1.875	99	2857793579
16	Nicotinic acid	C6 H5 N O2	124.03957	2.163	100	1037143853
17	Nicotinamide	C6 H6 N2 O	123.05553	2.324	99.9	3049318585
18	Adenosine 5'-monophosphate	C10 H14 N5 O7 P	348.07028	2.566	99.9	719844374.8
19	L-Isoleucine	C6 H13 N O2	132.10197	2.674	99.9	19076592592
20	Citric acid	C6 H8 O7	191.01868	2.676	98.7	63385345844
21	4-Oxoproline	C5 H7 N O3	128.03394	2.678	99.5	8189784682
22	D-(+)-Pyroglutamic Acid	C5 H7 N O3	130.05006	2.718	96.3	22679214012
23	Uracil	C4 H4 N2 O2	113.03495	3.606	99.3	1313390602
24	Alanyltyrosine	C12 H16 N2 O4	253.11824	4.293	91.2	487325839.3
25	Citraconic acid	C5 H6 O4	129.01796	4.355	99.8	2925087930
26	Adenosine	C10 H13 N5 O4	268.10379	4.614	100	8671532567
27	1,2,3-cyclopropanetricarboxylic acid	C6 H6 O6	173.00806	4.712	94	4783357736
28	2'-Deoxyadenosine	C10 H13 N5 O3	252.10912	4.861	100	579474404.3
29	Glycyl-L-leucine	C8 H16 N2 O3	189.12352	5.06	98.8	203921558.1
30	Prolylleucine	C11 H20 N2 O3	229.1546	5.195	94.6	187792450.7
31	L-Phenylalanine	C9 H11 N O2	166.0862	5.253	100	17474555402
32	Guanine	C5 H5 N5 O	152.05663	5.324	99.8	7251121211
33	3-(1-hydroxyethyl)-2,3,6,7,8,8a-hexahydropyrrolo[1,2-a]pyrazine-1,4-dione	C9 H14 N2 O3	199.10814	5.572	95.3	82705981.45

34	2'-O-Methyladenosine	C11 H15 N5 O4	282.12	5.95	99.3	1005820596
35	Methylsuccinic acid	C5 H8 O4	131.03378	6.001	97.9	189776049.4
36	1-butyl-2-methyl-4-nitro-1H-imidazole	C8 H13 N3 O2	206.09267	6.038	92.8	279382733.8
37	3,4-Dihydroxyphenylacetic acid	C8 H8 O4	167.03404	6.044	90.2	51232274.33
38	Thymidine	C10 H14 N2 O5	241.0827	6.485	96.3	248628138.2
39	N6-Me-Adenosine	C11 H15 N5 O4	282.11993	6.532	98.8	311444691.8
40	2,4-Xylidine	C8 H11 N	122.09666	6.883	97.4	18833975997
41	Leucylproline	C11 H20 N2 O3	229.15466	6.998	98.1	1799362832
42	4-Indolecarbaldehyde	C9 H7 N O	146.05995	7.345	96.8	1674991437
43	DL-Tryptophan	C11 H12 N2 O2	188.0704	7.349	99.9	20819630445
44	6-Methylindole	C9 H9 N	132.08081	7.352	96	454532034
45	N,N,4-Trimethylaniline	C9 H13 N	136.1123	8.089	98.7	1042821550
46	Neochlorogenic acid	C16 H18 O9	353.08762	8.139	96.5	2122522471
47	Caprolactam	C6 H11 N O	114.09161	8.243	99.9	30558089770
48	2,5-Dihydroxybenzaldehyde	C7 H6 O3	137.02321	8.309	98.2	262564618.9
49	(2R,3R)-2-(2,6-Dihydroxyphenyl)-3,5,7-trihydroxy-2,3-dihydro-4H-chromen-4-one	C15 H12 O7	305.06586	8.523	96.4	165671384.8
50	6-Methylquinoline	C10 H9 N	144.08109	8.623	97	590434114.5
51	2,3,4,9-Tetrahydro-1H-β-carboline-3-carboxylic acid	C12 H12 N2 O2	217.0975	8.623	99.8	774547916.9
52	Salicylic acid	C7 H6 O3	137.02321	8.985	98.9	98925402.89
53	Norharman	C11 H8 N2	169.07634	8.989	96.4	164679560.5
54	Gentisic acid	C7 H6 O4	153.0182	9.1	98.4	79817952.36
55	3-(propan-2-yl)-octahydropyrrolo[1,2-a]pyrazine-1,4-dione	C10 H16 N2 O2	197.12885	9.174	97.6	106206277.9
56	Resorcinol monoacetate	C8 H8 O3	151.03888	9.387	94.4	120012485.9
57	2-(2,6-dihydroxyphenyl)-3,5,7-trihydroxy-4H-chromen-4-one	C15 H10 O7	303.05038	9.403	99.2	178546689
58	Ferulic acid	C10 H10 O4	195.06552	9.554	96.4	152308525.9
59	Benzoic acid	C7 H6 O2	121.02821	9.595	99.6	174812302.6
60	Tolycaine	C15 H22 N2 O3	279.17056	9.6	96	260320810.8
61	Chlorogenic acid	C16 H18 O9	353.08752	9.963	99.6	4888586749
62	Phthaldialdehyde	C8 H6 O2	135.04439	10.117	98.7	527749831.5
63	Caffeic acid	C9 H8 O4	179.0341	10.133	97.5	366750047.5
64	5-(5-{5-[(2S)-1-(2-Fluorobenzyl)-2-pyrrolidinyl]-1,2,4-oxadiazol-3-yl}-2-pyridinyl)pyrimidine	C22 H19 F N6 O	425.1423	10.221	93.4	130937107.3
65	2-(Acetylamino)hexanoic acid	C8 H15 N O3	172.09695	10.353	97.6	62272046.61
66	2-Methylbenzoic acid	C8 H8 O2	135.0439	10.563	93.5	191797194.7
67	Fraxetin	C10 H8 O5	209.04466	10.684	92.9	185070531.8
68	Ageratriol	C15 H24 O3	235.16939	10.686	93.5	116976504.9
69	Cynaroside	C21 H20 O11	449.10809	10.705	97.6	326446049.8
70	Sibiricose A1	C23 H32 O15	593.17249	10.764	92.8	103173020.2

71	Cyclo(leucylprolyl)	C11 H18 N2 O2	211.14412	11.088	97.3	342474123.7
72	7-hydroxy-6-methoxy-2H-chromen-2-one	C10 H8 O4	193.04965	11.409	98.3	1736563955
73	Monocrotaline	C16 H23 N O6	326.15723	11.466	97.9	708630743.2
74	Lariciresinol 4-O-glucoside	C26 H34 O11	521.20288	11.493	96	56302140.09
75	Corymboside	C26 H28 O14	565.15533	11.642	93.8	2088922951
76	4-Hydroxycoumarin	C9 H6 O3	161.0233	11.695	94.9	286330877
77	7-Hydroxycoumarine	C9 H6 O3	163.03879	11.824	95.5	2134401164
78	2-Hydroxy-3-(5-hydroxy-7,8-dimethoxy-4-oxo-4H-chromen-2-yl)phenyl β-D-glucopyranoside	C23 H24 O12	493.13373	12.197	99.7	1057379957
79	Benzyl methacrylate	C11 H12 O2	159.08044	12.338	93.2	133936603.6
80	5-Hydroxy-2-(4-hydroxy-3-methoxyphenyl)-3,6-dimethoxy-4-oxo-4H-chromen-7-yl β-D-glucopyranoside	C24 H26 O13	523.14453	12.466	91.7	1133380785
81	Triethyl phosphate	C6 H15 O4 P	183.07808	12.481	99.7	337496280.2
82	N-(2,4-Dimethylphenyl)formamide	C9 H11 N O	150.09131	12.968	98.8	542556525.3
83	Dihexyl nonanedioate	C21 H40 O4	264.23199	13.043	99.9	301419511.5
84	Kaempferol	C15 H10 O6	287.05475	13.219	91.1	478733982.5
85	4,5-Dicaffeoylquinic acid	C25 H24 O12	515.11938	13.266	97.6	455070193.1
86	Azelaic acid	C9 H16 O4	187.09674	13.292	99.1	103058818.9
87	(3R)-8-hydroxy-3-(4-hydroxyphenyl)-3,4-dihydro-1H-2-benzopyran-1-one	C15 H12 O4	239.07018	13.65	94.7	112900316.4
88	Apigetrin	C21 H20 O10	433.11224	13.898	98.7	19299709429
89	Baicalin	C21 H18 O11	447.09134	14.245	99.8	1.54845E+11
90	Daidzin	C21 H20 O9	417.11758	14.664	96.5	881933155.6
91	5,7-dihydroxy-3,8-dimethoxy-2-phenyl-4H-chromen-4-one	C17 H14 O6	315.08597	14.848	97.3	1312016713
92	Nepetin	C16 H12 O7	317.06531	14.902	96.8	2920763093
93	6-O-Methylscutellarin	C22 H20 O12	477.10214	14.954	97.9	38756875577
94	Galangin	C15 H10 O5	271.05984	15.39	96.2	2741373567
95	5,7-dihydroxy-3-(4-hydroxyphenyl)-6-methoxy-4H-chromen-4-one	C16 H12 O6	301.07043	15.631	93.1	37550215251
96	Baicalein	C15 H10 O5	269.04532	15.759	99.8	28257539575
97	methyl 3-amino-4-(isopropylsulfonyl)-5-tetrahydro-1H-pyrrol-1-ylthiophene-2-carboxylate	C13 H20 N2 O4 S2	333.09671	15.772	94	4321377432
98	5,2'-Dihydroxy-6,7,8,6'-tetramethoxyflavone	C19 H18 O8	375.10696	15.947	96.8	1.05461E+11
99	Benzophenone	C13 H10 O	183.08058	16.055	99.8	424182105
100	5,6,7-trihydroxy-2-(4-methoxyphenyl)-4H-chromen-4-one	C16 H12 O6	301.07062	16.247	91.7	20561356118
101	(±)13-HpODE	C18 H32 O4	295.2269	16.377	93.8	321932288.3
102	(12Z)-9,10,11-trihydroxyoctade	C18 H34 O5	353.22955	16.5	99.3	793451103.4

	c-12-enoic acid					
103	Wogonin	C16 H12 O5	285.07526	16.637	99.8	81936045578
104	Atractylenolide III	C15 H20 O3	249.15	16.65	90.2	4022187946
105	Atractylenolide I	C15 H18 O2	231.14	16.65	90.4	13902126223
106	Chrysin	C15 H10 O4	253.05028	16.724	98	3311709870
107	5,7-dihydroxy-2-phenyl-4H-chromen-4-one	C15 H10 O4	255.06506	16.832	99.9	4515039087
108	Bis(4-ethylbenzylidene)sorbitol	C24 H30 O6	415.2113	17.051	99.5	1185582261
109	(1R,2R,6R,9R)-2,11,11-trimethyl-3-oxotricyclo[4.3.2.0Âa,â• µ]undecane-9-carboxylic acid	C15 H22 O3	233.1534	17.34	90.1	18118487242
110	(±)9-HpODE	C18 H32 O4	311.22287	17.87	96.3	395839547.4
111	Dipropyleneglycol dibenzoate	C20 H22 O5	365.13586	18.071	99.2	2330937372
112	7-Methyl-3-methylene-6-(3-oxobutyl)-3,3a,4,7,8,8a-hexahydro-2H-cyclohepta[b]furan-2-one	C15 H20 O3	231.138	18.103	94.1	1698902655
113	(±)9(10)-DiHOME	C18 H34 O4	313.23856	18.317	94.9	434698739.3
114	Dibutyl phthalate	C16 H22 O4	301.14096	18.404	98.9	2234314164
115	9-Oxo-10(E),12(E)-octadecadienoic acid	C18 H30 O3	295.22708	19.334	97.6	395195677.9
116	Docosahexaenoic acid	C22 H32 O2	341.26605	20.746	93.2	199050385.4
117	2,2'-Methylenebis(4-methyl-6-tert-butylphenol)	C23 H32 O2	339.23248	20.987	94.1	6352727416
118	4-tert-Amylphenol	C11 H16 O	163.11171	20.999	90.9	176626451.6
119	2-(14,15-Epoxyeicosatrienoyl) glycerol	C23 H38 O5	377.26608	21.289	91.2	451450723.1
120	octadec-9-ynoic acid	C18 H32 O2	263.237	21.289	96.8	125761767.3
121	(-)-Spiculisporic acid	C17 H28 O6	327.1814	21.352	92	464205552.5
122	Hexadecanamide	C16 H33 N O	256.26337	21.412	97.8	4527040787
123	Corticosterone	C21 H30 O4	391.21548	22.203	97	86548635.92
124	Palmitic Acid	C16 H32 O2	255.23285	22.482	99.7	365723199.2
125	Stearamide	C18 H37 N O	284.29446	22.492	99.1	34464127044
126	Di(2-ethylhexyl) phthalate	C24 H38 O4	391.284	22.852	99.4	707743358.1
127	1-Linoleoyl glycerol	C21 H38 O4	337.27362	22.889	98.4	1349734421
128	1-Stearoylglycerol	C21 H42 O4	381.29718	22.895	96.8	10476104829
129	Stearoyl Ethanolamide	C20 H41 N O2	310.3103	22.987	94.8	6159400867
130	Tridemorph	C19 H39 N O	298.31052	23.08	97.9	1166970600
131	Oleoyl ethanolamide	C20 H39 N O2	308.29459	23.754	99.2	881920851.3
132	Docosanamide	C22 H45 N O	340.35718	25.464	99	657912572.6

Table S2 The top 20 differentially expressed proteins between the Model group and the Control group, ranked by significance (log_2 Fold Change and adjusted P-values).

Number	Protein ID	**gene_name**	**log2 Fold Change**	**pvalue**
1	P62307	Snrpf	0.735522177	0.000974
2	P01863	Ighg	0.782408565	0.001294667
3	P51881	Slc25a5	0.669026766	0.0013585

4	Q8BSL7	Arf2	0.411426246	0.0013585
5	O35658	C1qbp	1.133125083	0.001474
6	O88587	Comt	1.883620816	0.001474
7	P26350	Ptma	1.61667136	0.001474
8	P32261	Serpinc1	0.367371066	0.001474
9	P61620	Sec61a1	0.385891154	0.001474
10	Q8BJ64	Chdh	-0.528378972	0.001474
11	Q99JX4	Eif3m	0.416839742	0.001474
12	P61164	Actr1a	0.778208576	0.001791
13	Q3U7R1	Esyt1	0.545968369	0.001856
14	Q9JIW9	Ralb	0.337235348	0.001856
15	O88487	Dync1i2	0.378511623	0.001959
16	E9Q414	Apob	2.046141782	0.006284667
17	Q60590	Orm1	-0.689659879	0.00703
18	Q3UMY5	Eml4	-0.957355663	0.008146
19	P09671	Sod2 Sod-2	-0.862496476	0.012633
20	Q62425	Ndufa4	-0.875671865	0.001959

Table S3 The top 20 differentially expressed proteins between the SA pairs group and the Model group, ranked by significance (log2 Fold Change and adjusted P-values).

Number	Protein	**gene_name**	**log2 Fold Change**	**P-value**
1	B2RXC1	Trappc11	1.871266686	0.017377
2	D3Z5S8	Tent5a Fam46a	-1.742724863	0.012363667
3	E9Q414	Apob	1.284587059	0.006284667
4	E9Q7G0	Numa1	0.845114398	0.011687333
5	E9Q912	Rap1gds1	1.029146346	0.031360667
6	G3X8U3	QNG1	-1.40599236	0.01592
7	G5E8Q8	Adgrf5	-1.117695043	0.0471265
8	O08585	Clta	-0.137503524	0.0061525
9	O08788	Dctn1	1.460634366	0.0127865
10	O08808	Diaph1	0.535331733	0.020471
11	O08810	Eftud2	0.542318163	0.007437667
12	P09671	Sod2	-0.811471031	0.012633
13	Q3UMY5	Eml4	-3.08236197	0.008146
14	O09159	Man2b1	0.415037499	0.013863667
15	O35114	Scarb2	-1.080657663	0.0063045
16	O35166	Gosr2	0.395928676	0.005455667
17	O35226	Psmd4	-0.63691458	0.006172667
18	O35381	Anp32a	-0.214124805	0.008430667
19	O35566	Cd151	0.257275137	0.008791
20	O35658	C1qbp	-0.754174884	0.001474
